# Potential impact of stress activated retrotransposons on genome evolution in a marine diatom

**DOI:** 10.1186/1471-2164-10-624

**Published:** 2009-12-22

**Authors:** Florian Maumus, Andrew E Allen, Corinne Mhiri, Hanhua Hu, Kamel Jabbari, Assaf Vardi, Marie-Angèle Grandbastien, Chris Bowler

**Affiliations:** 1 CNRS UMR8186, Biologie Moléculaire des Organismes Photosynthétiques, Ecole Normale Supérieure, 46 rue d'Ulm, 75230 Paris cedex05, France; 2 J. Craig Venter Institute, 10355 Science Center Drive, San Diego, CA 92121, USA; 3 Laboratoire de Biologie Cellulaire, Institut Jean-Pierre Bourgin, INRA Versailles-Grignon, 78026 Versailles, France; 4 Environmental Biophysics and Molecular Ecology Group, Institute of Marine and Coastal Sciences, Rutgers University, 71 Dudley Road, New Brunswick, NJ 08901, USA; 5 Stazione Zoologica 'Anton Dohrn,' Villa Comunale, I-80121 Naples, Italy

## Abstract

**Background:**

Transposable elements (TEs) are mobile DNA sequences present in the genomes of most organisms. They have been extensively studied in animals, fungi, and plants, and have been shown to have important functions in genome dynamics and species evolution. Recent genomic data can now enlarge the identification and study of TEs to other branches of the eukaryotic tree of life. Diatoms, which belong to the heterokont group, are unicellular eukaryotic algae responsible for around 40% of marine primary productivity. The genomes of a centric diatom, *Thalassiosira pseudonana*, and a pennate diatom, *Phaeodactylum tricornutum*, that likely diverged around 90 Mya, have recently become available.

**Results:**

In the present work, we establish that LTR retrotransposons (LTR-RTs) are the most abundant TEs inhabiting these genomes, with a much higher presence in the *P. tricornutum *genome. We show that the LTR-RTs found in diatoms form two new phylogenetic lineages that appear to be diatom specific and are also found in environmental samples taken from different oceans. Comparative expression analysis in *P. tricornutum *cells cultured under 16 different conditions demonstrate high levels of transcriptional activity of LTR retrotransposons in response to nitrate limitation and upon exposure to diatom-derived reactive aldehydes, which are known to induce stress responses and cell death. Regulatory aspects of *P. tricornutum *retrotransposon transcription also include the occurrence of nitrate limitation sensitive *cis*-regulatory components within LTR elements and cytosine methylation dynamics. Differential insertion patterns in different *P. tricornutum *accessions isolated from around the world infer the role of LTR-RTs in generating intraspecific genetic variability.

**Conclusion:**

Based on these findings we propose that LTR-RTs may have been important for promoting genome rearrangements in diatoms.

## Background

Transposable elements (TEs) are mobile genetic sequences found within the genomes of most organisms. Sequences derived from TEs represent a genomic fraction of 3% in baker's yeast [1], ~20% in fruit fly [2-4], 45% in human [5,6] and over 80% in maize [7,8]. They are thought to be important contributors to genome evolution by inserting into genes or genetic regulatory elements, thereby disrupting gene function, altering levels of gene expression, triggering chromosomal rearrangements, and adding to or subtracting from the physical size of a host genome [9]. TEs are classified into two groups based on their mode of transposition: retrotransposons or Class 1 TEs which replicate through reverse transcription of an mRNA intermediate, and DNA transposons or Class 2 TEs that use a "cut and paste" mechanism.

A pervasive group of retrotransposons are those flanked by long terminal repeats (LTRs), also typical of retroviruses to which they are related. The LTR direct sequence repeats flank the internal region that encodes both structural and enzymatic proteins with homology to the GAG and POL proteins of retroviruses. The *gag *gene encodes structural proteins that form the virus-like particle (VLP), inside which reverse transcription takes place. The *pol *gene encodes several enzymatic functions, including a protease (PR) that cleaves the POL polyprotein, a reverse transcriptase (RT) that copies the retrotransposon RNA into cDNA, a ribonuclease H domain (RH), and an integrase (IN) that integrates the cDNA into the genome. Two main groups of LTR retrotransposons (LTR-RTs) are found throughout eukaryotes, and are distinguished by the organization of their *pol *genes and similarities among their encoded RT proteins [10]. These groups are referred to as *Ty1/copia *elements (Pseudoviridae) and *Ty3/gypsy *elements (Metaviridae), which respectively display a PR, IN, RT, RH and PR, RT, RH, IN gene organization.

The unicellular chlorophyll *c*-containing algal class Bacillariophyceae (diatoms) is among the most successful and diversified groups of photosynthetic eukaryotes, with possibly over 100,000 extant species [11] widespread in all kinds of humid and open water environments. The contribution of diatom photosynthesis to marine primary productivity has been estimated to be around 40% [12,13]. Diatoms have a peculiar genetic makeup because they are likely to have emerged following a secondary endosymbiotic process between a photosynthetic eukaryote, most probably red algal-like, and a heterotrophic eukaryote [14]. They are traditionally divided into two orders: the centric diatoms which are radially symmetrical and are thought to have arisen around 180 Million years ago (Mya), followed by the pennate diatoms around 90 Mya which are bilaterally symmetrical. Genome sequences of the centric diatom *Thalassiosira pseudonana *and the pennate diatom *Phaeodactylum tricornutum *have recently become available [15,16]. Because diatoms are single celled organisms that typically reproduce mitotically, the activity of LTR-RTs might have particularly profound effects on genome evolution since any non-lethal retroelement insertion will be transmitted to subsequent generations.

In an analysis of the *T. pseudonana *genome, Armbrust and collaborators identified several TEs [15]. In the current work, we have identified additional TEs in both diatom genomes and we show that LTR-RTs are the most abundant elements, particularly in *P. tricornutum *where they have amplified enormously. Phylogenetic analysis of the RT domain shows that diatom *Ty1/copia*-like elements belong to different lineages, and that two of them are diatom specific. Examination of the CAMERA metagenomic database reveals that these elements are also widespread in different oceans. The potential ecological relevance of these elements for driving genome and population evolution and heterogeneity has been assessed by examining their expression in response to stress as well as their distribution in *P. tricornutum *accessions collected from different locations worldwide. We also examine whether or not *cis*-regulatory elements within LTR sequences contain sufficient information for driving retrotransposon transcription in response to nitrate deprevation and if alterations in cytosine methylation play a role in retrotransposon expression.

## Results

### Expansion of LTR Retrotransposons in the *P. tricornutum *genome

**Figure 1:**
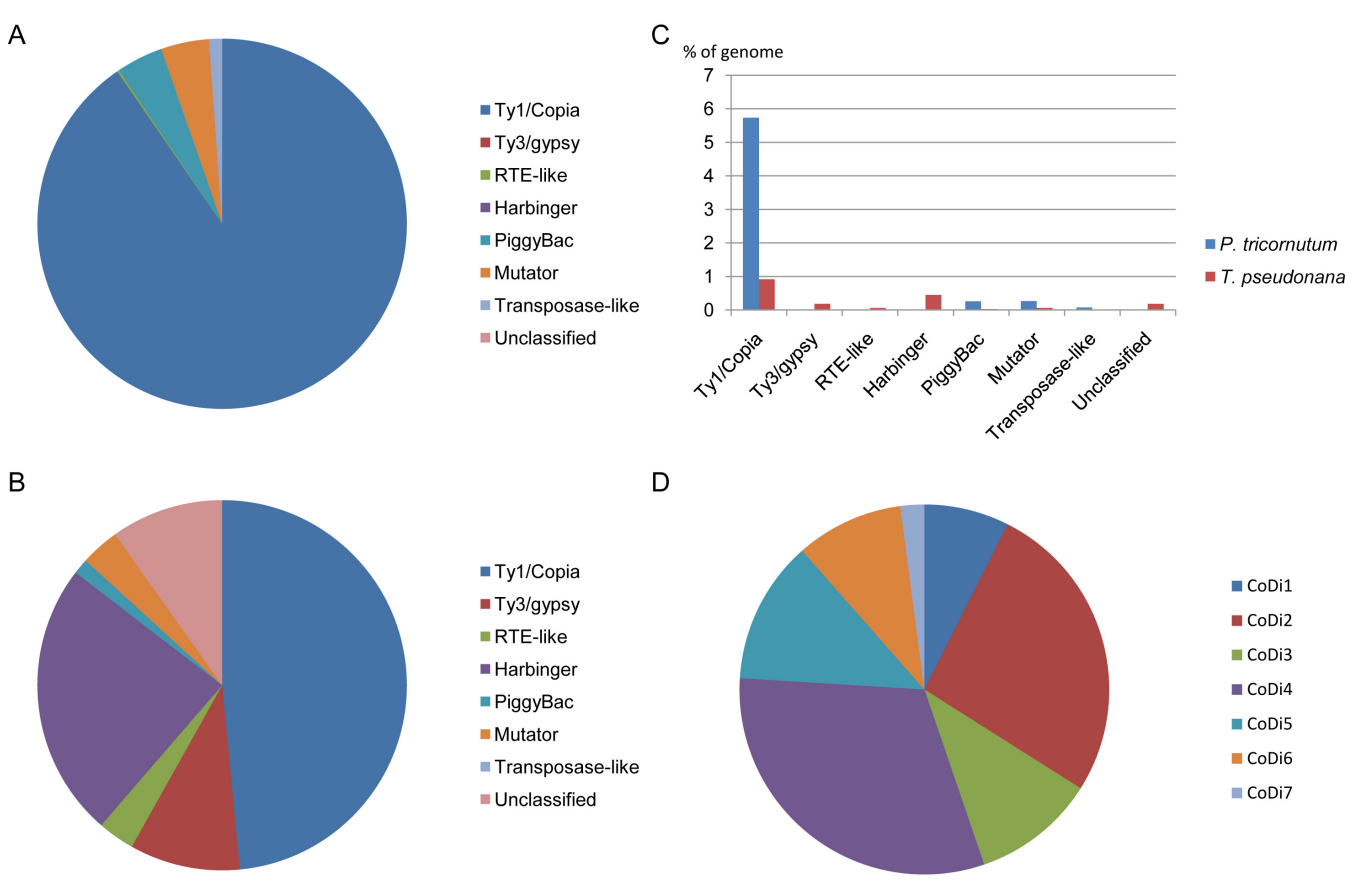
**Composition of the TE complements in the *P. tricornutum *and *T. pseudonana *genomes**. (A and B) Pie chart representing the relative abundance of different TEs to the *P. tricornutum *(A) and *T. pseudonana*(B) TE complements. (C) Histogram representing percent genome coverage across the diatom TE complements. (D) Pie chart representing the relative contribution of the different CoDi groups to the *P. tricornutum *LTR-RT complement.

We first examined the TE content of diatom genomes. In the *T. pseudonana *genome Armbrust and collaborators identified some *Ty1/copia *and *Ty3/gypsy*-like elements, a family of RTE-like non-LTR retrotransposons, Mutator-like (here denoted as TpMuDR1) and *Harbinger*-like DNA transposons, as well as some unknown unclassified repeated sequences [15,17]. In the present work, we could identify additional LTR-RT elements in the *T. pseudonana *genome (Figure 1). We also identified numerous *Ty1/copia*-like elements in the *P. tricornutum *genome as well as an RTE-like element, two distinct families of *Mutator*-like elements (one being closely related to *TpMuDR1 *elements), and two other different types of uncharacterized transposase-containing elements (one being weakly related to *piggyBac *transposons and for which we also found a homolog in the *T. pseudonana *genome (see Materials and Methods). *Ty3/gypsy*-like elements were not found in the *P. tricornutum *genome (Figure 1A).

To analyse the contribution of TEs to diatom genomes we used the diatom TE DNA sequences to run the RepeatMasker program [18] on both genomes. In total, we found that TEs contribute 1,665 kb (6.4%) of the *P. tricornutum *genome and 590 kb (1.9%) of the *T. pseudonana *genome. Of these, LTR-RTs are the most abundant in both genomes and constitute 90% and 58% of the *P. tricornutum *and *T. pseudonana *TE complement, respectively (Figure 1A and 1B). Harbinger elements also appear to represent a significant proportion in *T. pseudonana*. In total, the RepeatMasker output indicated that sequences deriving from LTR-RTs make up 5.8% of the *P. tricornutum *genome [16] and 1.1% of the *T. pseudonana *genome [15] (Figure 1C). It thus appears that Ty1/copia-like LTR-RTs have significantly expanded in the *P. tricornutum *genome.

### Classification of LTR retrotransposon sequences

To further investigate the diatom LTR-RT elements, we manually screened the *P. tricornutum *and *T. pseudonana *nuclear genomes for the presence of putatively autonomous LTR-RTs (see Materials and Methods), and found a total of 42 and 13 putative active elements in the final unmasked assemblies of the *P. tricornutum *and *T. pseudonana *nuclear genomes, respectively. Most of these have greater than 95% identical LTR pairs and display only one or no stop codon/frameshifts between the *gag *and *pol *genes (Additional file 1 and Materials and Methods). All the selected sequences from *P. tricornutum *and 11 from *T. pseudonana *belonged to the *Ty1/copia *class with *pol *domains ordered as expected (PR, IN, RT, RH), and the two remaining sequences from *T. pseudonana *belonged to the *Ty3/gypsy *class with *pol *domains also ordered in a typical fashion (PR, RT, RH, IN).

The 53 *Ty1/copia*-like elements identified in the *P. tricornutum *and *T. pseudonana *genomes were classified on the basis of RT domain sequence similarity (see Materials and Methods). Seven groups of *Ty1/copia*-like retroelements were identified and denoted CoDi1 to CoDi7 (*Ty1/Copia*-like elements from Diatoms) (Additional file 1, Figure 1). While the CoDi1 to CoDi5 groups are quite homogeneous, the CoDi6 group consists of a set of diverse elements. The CoDi7 group is composed of a single element from *P. tricornutum *(PtC47). The CoDi1-2-3-7 groups are specific to *P. tricornutum *whereas the CoDi4-5-6 groups are composed of elements found in both diatom genomes. It appears that the CoDi2 and CoDi4 groups are major components of LTR-RT expansion in the *P. tricornutum *genome (Figure 1D).

### Phylogenetic analysis

**Figure 2:**
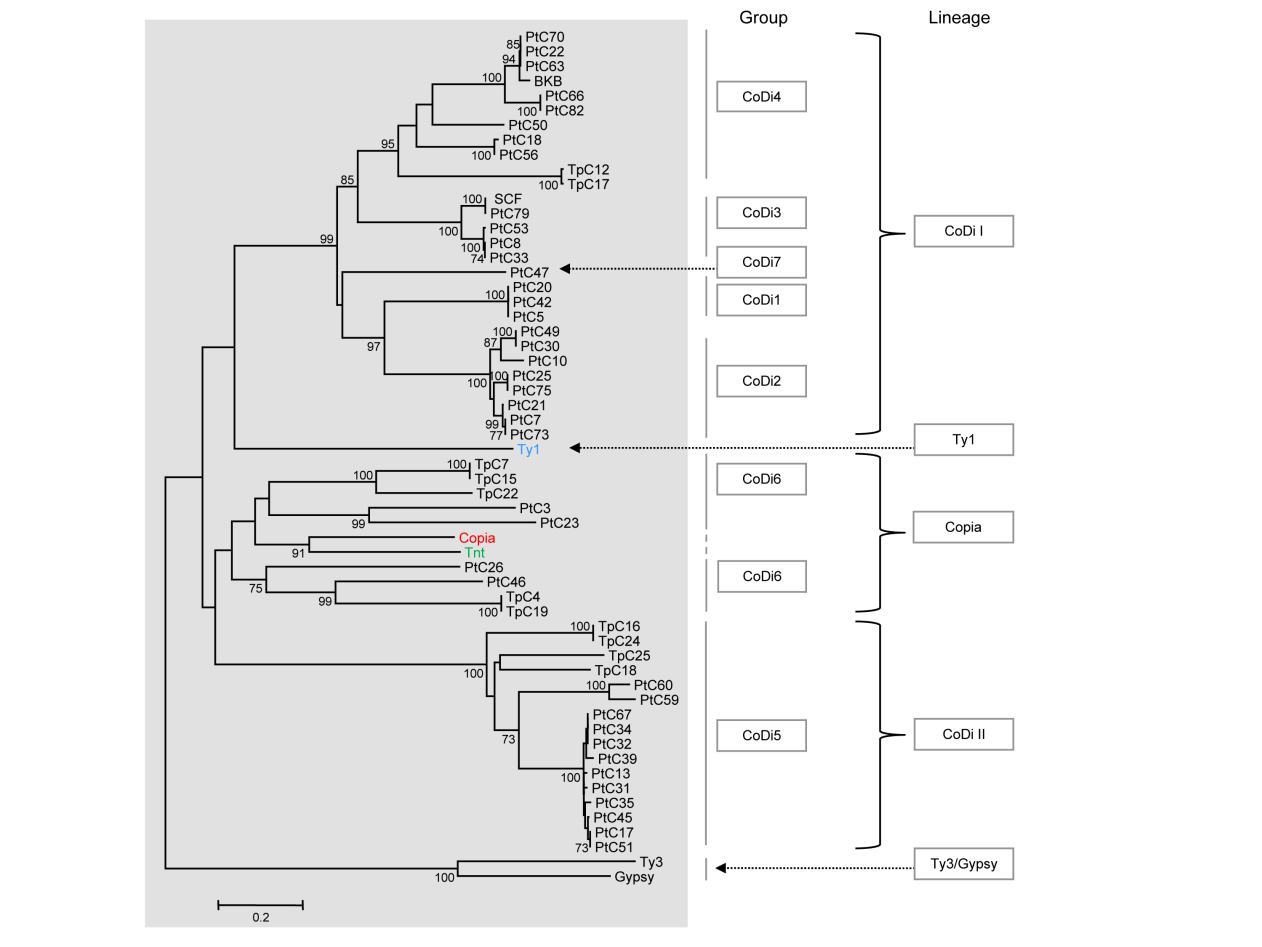
**Phylogenetic tree showing the relationships between the CoDis and other *Ty1/copia*-like elements**. This tree uses the RT domains from *Ty3 *and *gypsy *as outgoup and was constructed with the NJ method with the MEGA4 software [54]. The bootstrap values were calculated over 1,000 iterations and bootstrap scores over 70% are shown.

We constructed a phylogenetic tree from a CLUSTALW multiple alignment of the RT domains from the *Ty1/copia*-like shown in Additional file 1 as well as reference sequences for the Ty1 and Copia lineages (*Tnt *from tobacco, *copia *from fruit fly, and *Ty1 *from budding yeast) (Figure 2). We observed a distribution of sequences into seven clusters corresponding to the groups defined previously (Additional file 1). The most homogeneous clusters represent the groups CoDi1-2-3 composed of sequences present only in *P. tricornutum*. The PtC47 element representing the CoDi7 group appears distantly linked to the CoDi1-2 groups. The lineage linking the CoDi1-2-3-4-7 groups was denoted CoDiI (Figure 2). Like CoDi4, the CoDi5 group is composed of sequences from both the centric and the pennate diatom and constitutes a separate lineage we called CoDiII. Finally, the elements from the CoDi6 group which includes elements from both genomes cluster into a highly heterogeneous lineage together with the marker elements *Tnt *and *copia*. In this tree, we can therefore recognize a class of diatom *Ty1/copia*-like elements most closely related to known elements from the *Copia *lineage as well as two diatom-specific lineages, CoDiI and CoDiII (Figure 2).

**Figure 3:**
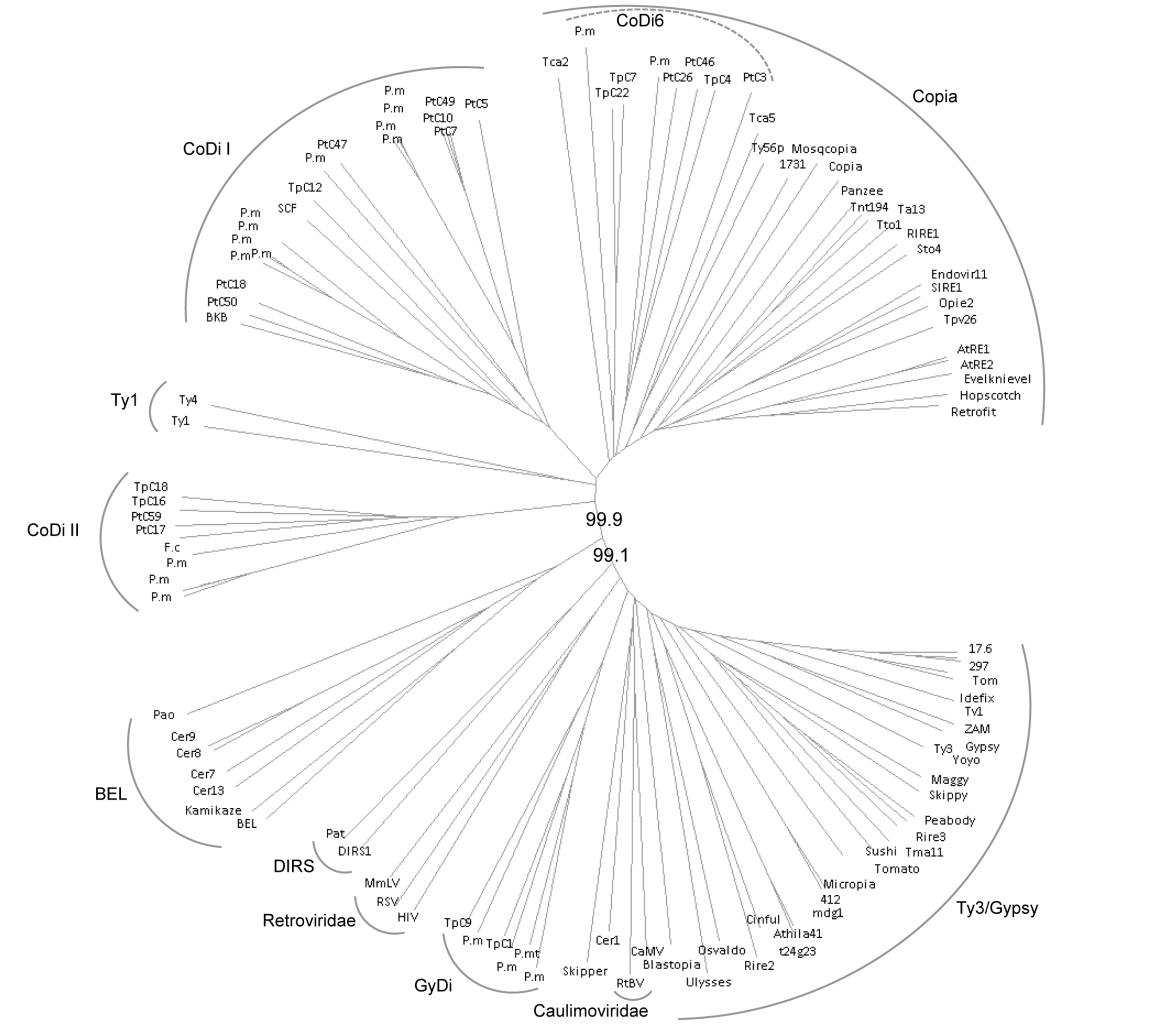
**Phylogenetic tree showing the relationships between CoDis and other LTR-RT and retroviral lineages**. The bootstrap values were calculated over 1,000 iterations and are indicated for two basal nodes. The tree was constructed with the NJ method using the SplitsTree4 software [55]. Species abbreviations: P. mt (*Pseudonitzschia multistriata*); P. m (*Pseudonitzschia multiseries*); F. c (*Fragilariopsis cylindrus*).

To better clarify the evolutionary relationships between the LTR-RTs from diatoms and other retrotransposable elements, we studied RT sequences from a representative subset of elements from each CoDi group defined on the basis of our previous analysis (Additional file 1 and Figure 2) and RT sequences that we identified from the pennate diatoms *Fragilariopsis cylindrus; Pseudo-nitzschia multistriata*, and *Pseudo-nitzschia multiseries*. A phylogenetic representation of diatom RT domains with those belonging to the major lineages of LTR retrotransposons and retroviruses (see Materials and Methods) showed that the heterogeneous CoDi6 group appears closest to the major Copia lineage (Figure 3), which includes sequences from animals, plants, yeast, and heterokonts (diatoms), which confirms the origin of the Copia lineage as deeply rooted in eukaryotes. This tree also confirms the distant evolutionary relationships that link the elements from the CoDiI lineage to the Ty1 and Copia lineages and the even more distant relationships that link the CoDiII lineage to these other elements. We also note that the RT sequences from the other diatoms cluster in the CoDiI, CoDiII and Copia lineages, and that the *Ty3/gypsy*-like elements from *T. pseudonana, P. multiseries *and *P. multistriata *also segregate together in a diatom-specific cluster (Figure 3).

### Expression of LTR retrotransposons in *P. tricornutum *and *T. pseudonana*

**Figure 4:**
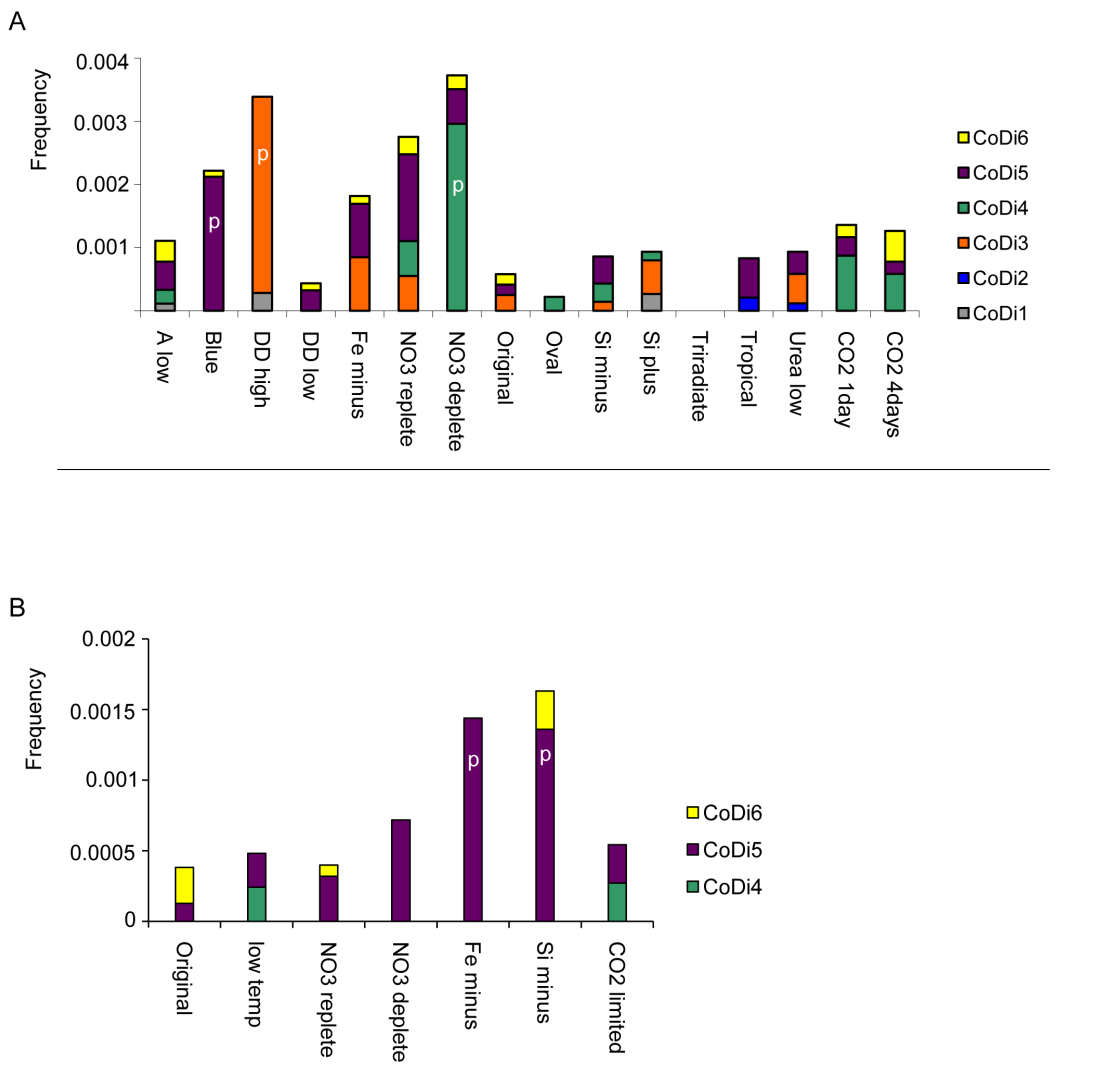
**Abundance of CoDi-encoding ESTs in different conditions**. (A) EST frequencies of the *P. tricornutum *CoDi elements listed in Additional file 1 within the 16 *P. tricornutum *cDNA libraries described and available at http://www.biologie.ens.fr/diatomics/EST3/. CoDi7 group does not have any EST support. (B) EST frequencies of the *T. pseudonana *CoDi elements listed in Additional file 1 within the 7 *T. pseudonana *cDNA libraries described and available at http://www.biologie.ens.fr/diatomics/EST3/. (A and B) Letter p indicates statistically-supported a (Pearson's Chi squares p = 0.0000) higher EST frequency of a CoDi group in this condition respect to the original library (non-stressed).

To examine TE expression, the complete nucleotide sequences of the full length elements from *P. tricornutum *listed in Additional file 1 were searched in the diatom digital gene expression database (available at http://www.biologie.ens.fr/diatomics/EST3/) [19] using BLAST. This database comprises more than 200,000 ESTs from *P. tricornutum *and *T. pseudonana *cells grown in a range of different conditions, many of which correspond to different abiotic stresses. The global EST profile of each CoDi group reveals a pattern of higher expression levels under some stress conditions (Figure 4). In particular, we focused on two *P. tricornutum *CoDi1 lineage elements that were strongly induced under conditions of nitrate starvation and following exposure to the toxic reactive aldehyde decadienal (DD) (Figure 4). These were denoted *Blackbeard *(*Bkb*) and *Surcouf *(*Scf*), respectively, and the contribution of CoDi3 and CoDi4 to the nitrate deplete and DD high libraries are due exclusively to these elements. qRT-PCR was subsequently used to confirm their upregulation in response to nitrogen starvation and following exposure to DD (Table 1).

**Table 1:** 

Treatment	Target Element	Reference Gene	2^-ΔΔCT^(fold change)*
DD2 2 hrs	*Surcouf*	TBP	62.83 (38.35-102.94)
DD2 6 hrs	*Surcouf*	TBP	106.64 (78.14-145.54)
DD2 30 hrs	*Surcouf*	TBP	26.48 (15.27-45.92)
DD2 4 days	*Surcouf*	TBP	2.27 (1.56-3.28)
24 hrs (N limitation)	*Blackbeard*	18S rDNA	3.51 (2.67-4.61)
2 weeks (N limitation)	*Blackbeard*	18S rDNA	92.21 (61.59-138)

*Fold change with respect to expression levels in untreated cultures

### Regulation of *Blackbeard*

**Figure 5:**
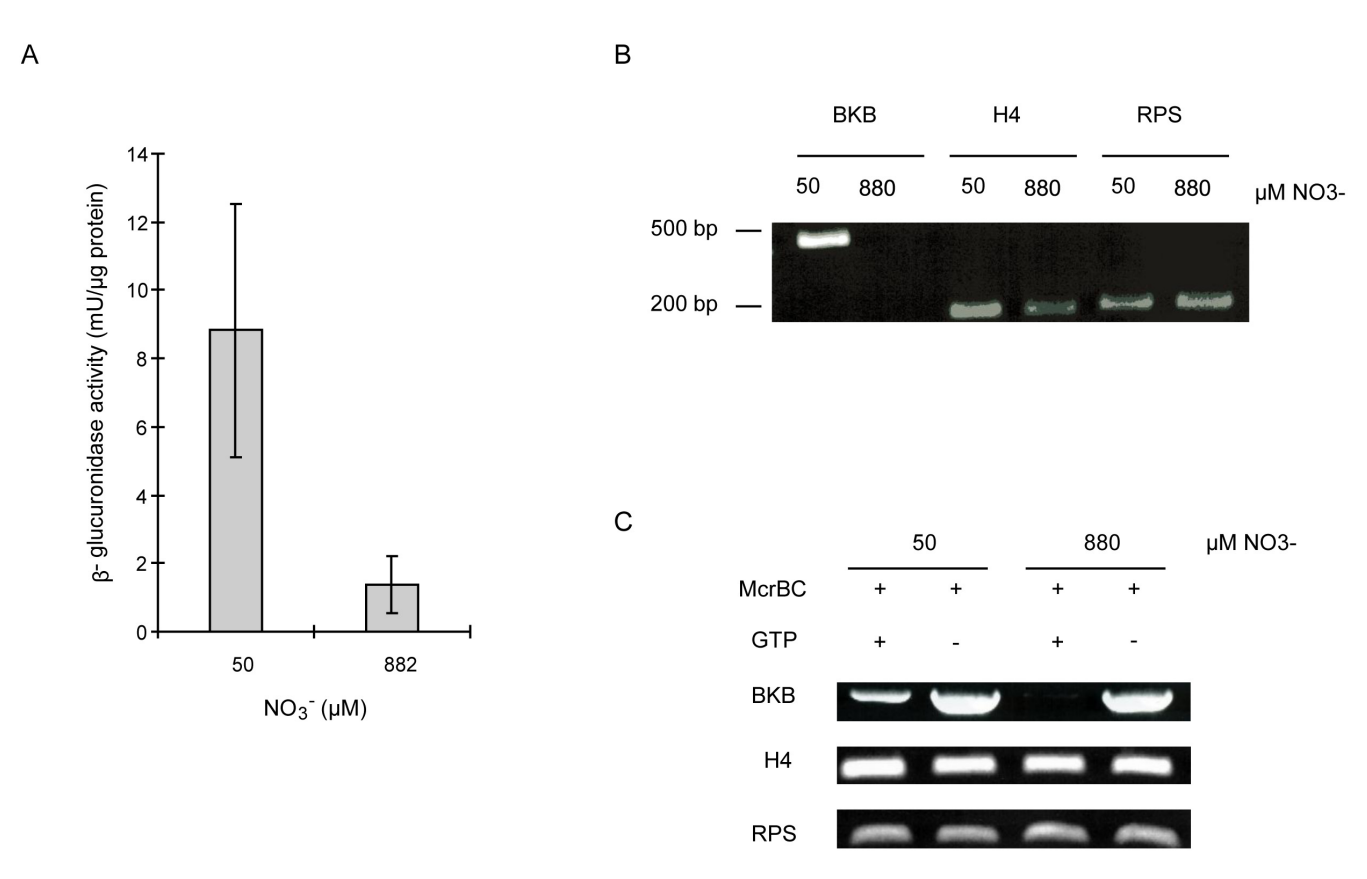
**Regulation of *Blackbeard *expression**. (A) Effect of nitrate limitation on the expression of the pLTRbkb-GUS-FcpA construct in transgenic *P. tricornutum *cells. Data represent the average with standard error from seven independent cultures after two weeks nitrate limitation (50 μM NO3-) compared to standard growth medium (882 μM NO3-). (B) Verification of *Blackbeard *transcriptional activation by semi-quantitative RT PCR in the cultures used for McrPCR. (C) McrPCR on *Blackbeard *and H4 and RPS controls using DNA extracted from *P. tricornutum *cells grown under normal and nitrate-limited conditions.

In an effort to better understand *Blackbeard *expression in response to nitrogen limitation, we examined its transcriptional and chromatin-level regulation. Because *cis*-acting elements regulating LTR-RT expression are typically found within LTRs [20,21], we generated a construct containing the *Blackbeard *LTR fused to the *β-glucoronidase *(GUS) reporter gene. Although the *Blackbeard *LTR is only 163 bp, spectrophotometric GUS measurements on *P. tricornutum *lines transformed with this construct showed that it was sufficient to activate transcription in response to nitrate starvation (Figure 5A). This shows that the *Blackbeard *LTR alone contains sufficient *cis*-regulatory element information to drive *Blackbeard *expression in response to nitrate limitation.

Cytosine methylation is commonly found in the DNA sequences of transposable elements (at least in genomes in which methylation occurs) and is thought to be involved in the heterochromatin formation and maintenance that controls TE mobility. TE mobilization has been shown to be associated with DNA hypomethylation [22-24], and hypomethylation has also been found to accompany active transposition in response to stress [25,26]. We therefore assessed whether the *Blackbeard *element was methylated using McrPCR. In this method, DNA is digested with McrBC which cleaves DNA containing methylcytosine. Consequently, PCR using McrBC-digested DNA as template leads to a decrease of amplification at methylated (cut) loci with respect to untreated DNA. We first observed that all LTR-RTs tested were methylated in the *P. tricornutum *genome under normal growth conditions (data not shown), demonstrating that DNA methylation does occur in this diatom. We then compared McrPCR amplification levels using DNA extracted from *P. tricornutum *cells grown in normal and nitrate limiting conditions. Figures 5B and 5C show that the induction of *Blackbeard *in response to nitrate limitation was accompanied by a decrease in cytosine methylation, suggesting that chromatin remodeling occurs at the *Blackbeard *locus in response to nitrate limitation. Preliminary results from bisulfite sequencing indicate that methylation at the *Blackbeard *locus occurs in a CpG context (data not shown).

### Insertion polymorphism between *P. tricornutum *accessions

**Figure 6:**
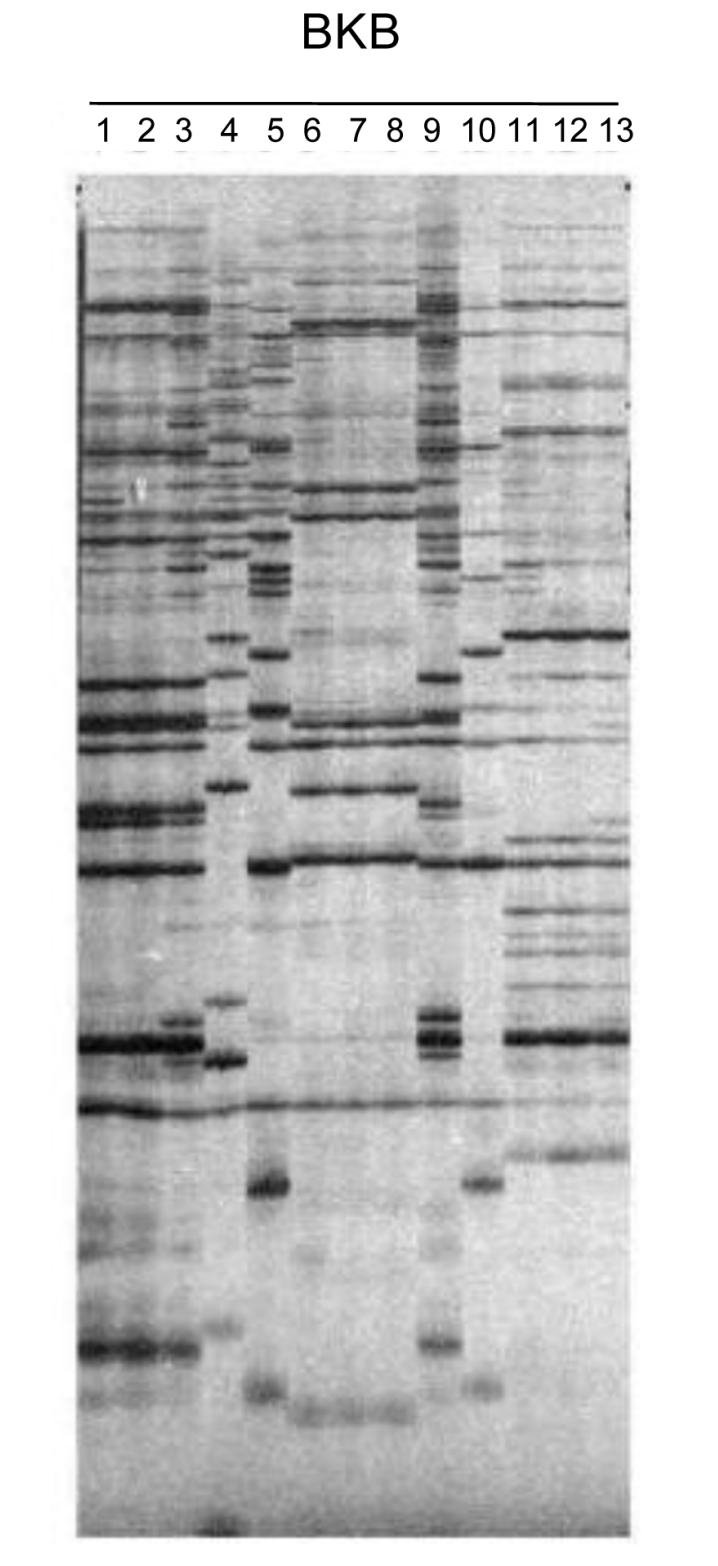
**Sequence Specific Amplified Polymorphism analysis of *Bkb *in 13 *P. tricornutum *accessions**. Each amplified insertion is revealed as a band on a sequencing gel and genomic DNA from the different accessions produces a characteristic fingerprint of bands.

Although suggestive, the induction of *Bkb *expression by nitrate limitation is not proof that it can actually drive genome rearrangements by *de novo *insertion in the genome. In order to better evaluate this possibility, we assessed the distribution of *Bkb *elements in thirteen *P. tricornutum *accessions collected from different locations worldwide by Sequence Specific Amplified Polymorphism (SSAP) [27] (see Materials and Methods). SSAP amplifies the region between a PCR primer site near the end of an element and an adjacent restriction site in the flanking genomic DNA. This global analysis revealed clear differences in *Bkb *insertion profiles in different accessions, demonstrating that it has been transposing in natural environments (Figure 6). We were able to confirm the same phenomenon with two other elements, *Scf *and *PtC34 *(data not shown). We subsequently cloned several bands from the SSAP gel in order to determine some insertion sites in accessions other than the sequenced genotype (Additional file 2). None of the sequences we obtained were inserted inside genes, and most were inserted into intergenic regions, sometimes very close to coding sequences. For example, a PtC34 insertion found in Pt6-7-8 is located 82 bp upstream of the 5' UTR of the gene encoding uroporphyrinogen-III synthase, which catalyses the sixth step of heme biosynthesis. We also found several sequences corresponding to *Bkb *and *Scf *inserted into other TEs (Additional file 2).

### Two distinct haplotypes at loci containing TEs

**Figure 7:**
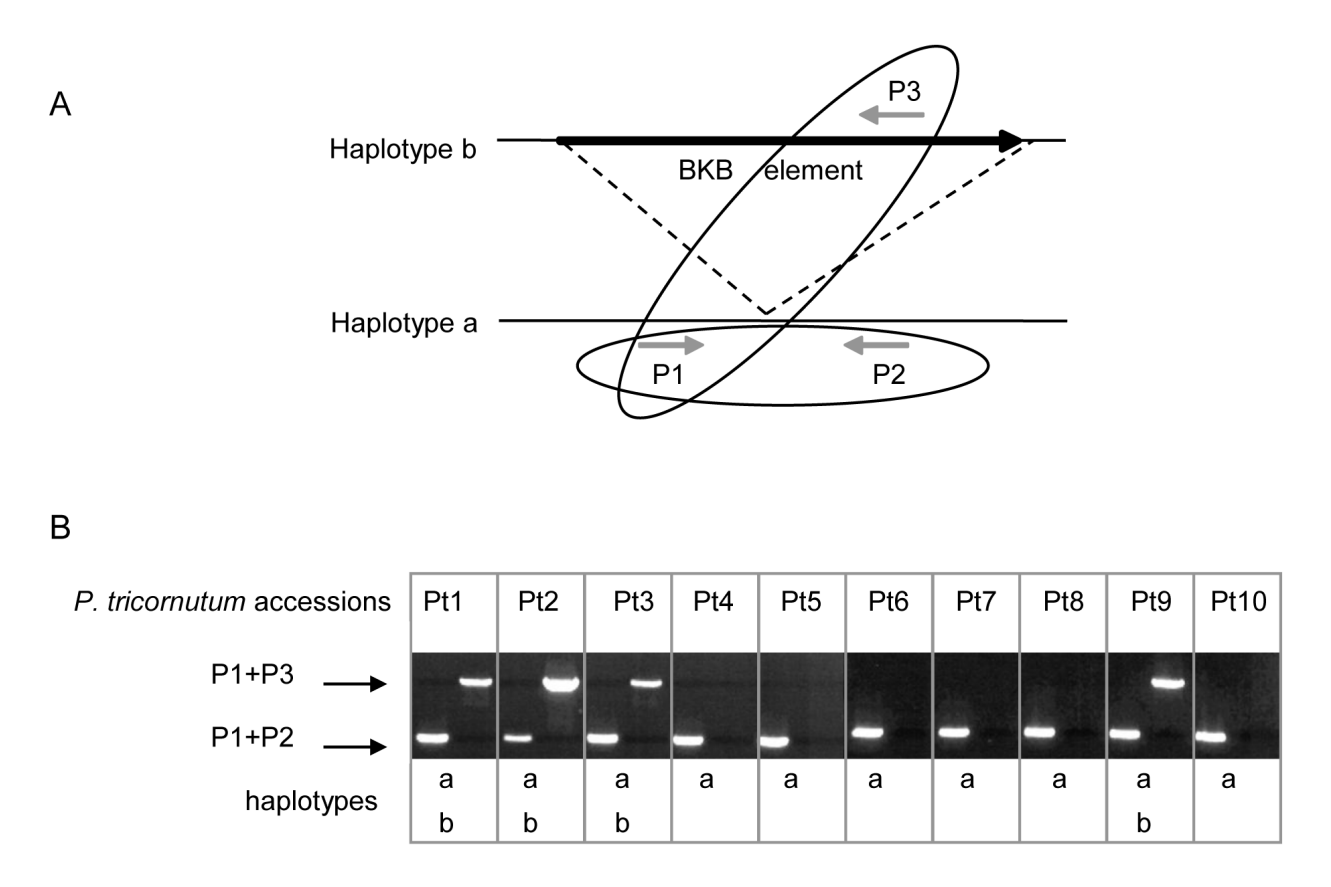
**Analysis of the *Blackbeard *locus**. (A) Schematic representation of the primer pairs used to perform PCR at the *Blackbeard *locus. Primer pairs are embedded within ovals and dashed lines indicate the projection of the *Bkb *locus found in haplotype b to its native target site on haplotype a. (B) Haplotype analysis by PCR to assess the presence/absence of the *Blackbeard *insertion in ten *P. tricornutum *accessions. Haplotypes a and b respectively refer to the absence and presence of the *Blackbeard *insertion.

Analysis of sequencing reads around several TE insertion sites revealed that many were inserted in just one of the haplotypes and that the other haplotype was apparently intact. As an example, the *Blackbeard *insertion is shown in Figure 7A and Additional file 3. For this (and all other) insertion events, we could verify by PCR that the allelic specificity is conserved in all accessions in which they are found (accessions Pt1, Pt2, Pt3, and Pt9 for *Bkb*), whereas in the other accessions we could only detect the empty locus (Figure 7). Because the oldest of these accessions was collected more than one hundred years ago [27], we can conclude that the *Bkb *insertion must have occurred before this time.

### TE-mediated recombination in the *P. tricornutum *genome

To shed light on the potential impact of LTR-RTs on genome dynamics, we analyzed some signatures of intra- or inter-element recombination in the *P. tricornutum *and *T. pseudonana *genomes [28]. Unequal intrastrand homologous recombination between LTRs of different elements belonging to the same family is a typical example and can result in a net loss of the DNA in between the elements involved. Five examples of this were found in our study of the *P. tricornutum *genome, all of which resulted in clearly recognizable recombinant products in which apparently intact elements with more than 99% identical LTRs lacked the target-site duplication (TSD) (see Additional file 1) and were therefore expected to be the product of homologous recombination between two family members. On the other hand, we found no example of this kind in the *T. pseudonana *genome.

**Figure 8:**
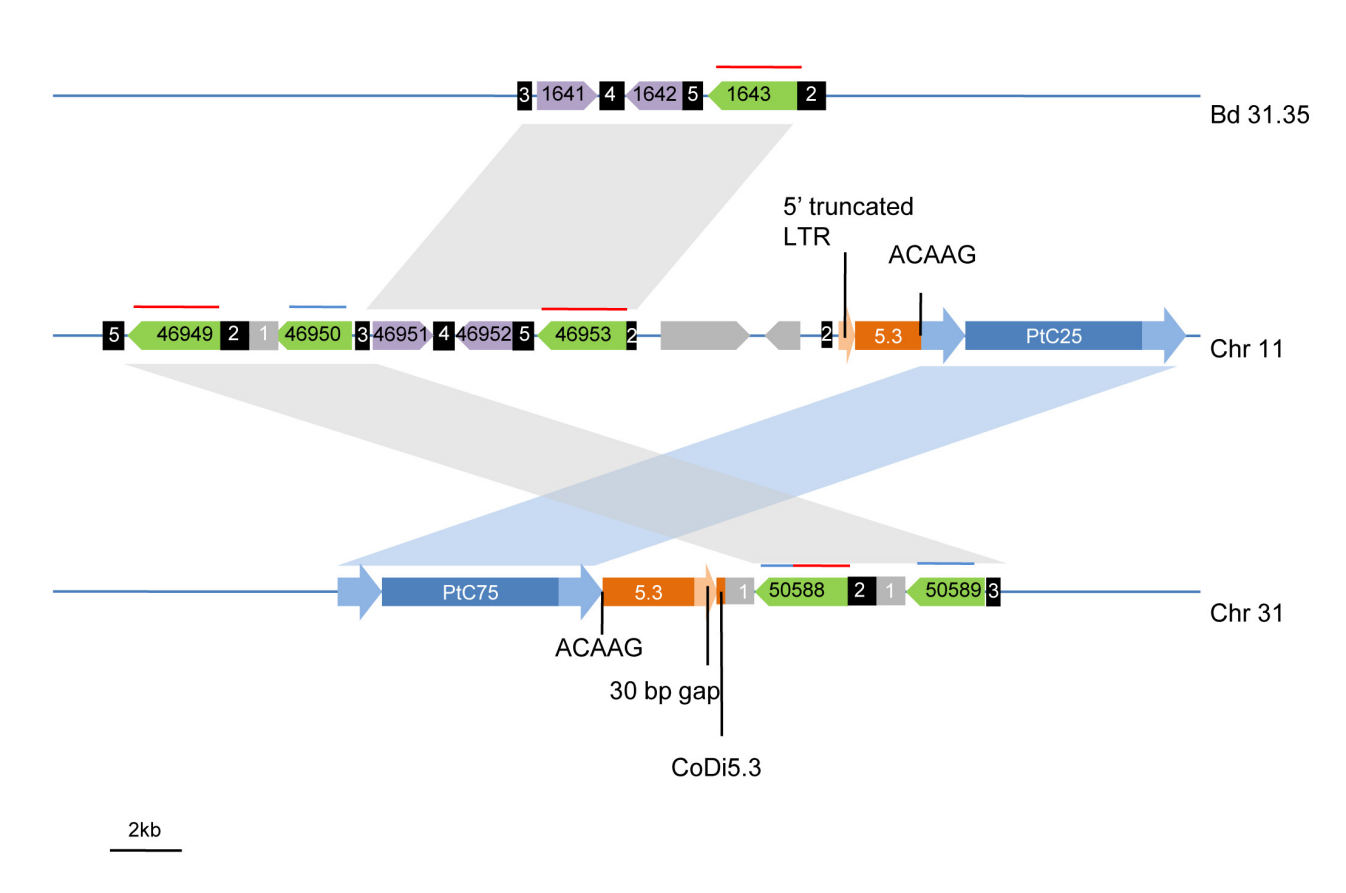
**Schematic representation of the PtC25 and PtC75 recombinant loci**. LTR-RT of the CoDi5.3 (orange) and CoDi2.3 (blue) groups are drawn with their LTRs (flanking arrows). Gene family 1 (green) and gene family 2 (purple) and other genes (grey) are drawn as arrows. Gene family 1 is further distinguished by red and/or blue bar on top and similar colors indicate similar sequences (see Additional file 4). Black or grey boxes with identical numbers indicate similar intergenic regions. Grey parallelograms project large duplicated regions from chromosome to chromosome. The blue parallelogram indicates the high similarity between the PtC25 and PtC75 elements. We indicate a 30 bp gap found in the CoDi5.3 segment flanking PtC75. We also indicate that the PtC25-associated CoDi5.3 entity contains a 5' truncated LTR which starts precisely where the gap described on chromosome 31 ends, further consolidating the historical link between these two loci. Bd 31.35 indicates a scaffold that could not be successfully mapped during *P. tricornutum *genome assembly.

We also noticed that the two elements constituting the CoDi2.3 family, PtC25 (on chromosome 11) and PtC75 (on chromosome 31), both lacked a TSD (Additional file 1). Closer examination of these loci revealed evidence that these two elements have been co-involved in a recombination event (Figure 8). Specifically, we found that the 5' flanking region of PtC25 consists of a truncated CoDi5.3 element and that the 3' flanking sequence of PtC75 also consists of a truncated CoDi5.3 which is the exact continuation of the PtC25-flanking entity but in addition contains a duplication of an ACAAG motif. The most parsimonious explanation for this organization is that either PtC25 or PtC75 inserted inside a CoDi5.3 element and that this insertion generated duplication of the target site (ACAAG). Subsequently, PtC25 and PtC75 engaged in a recombination event that split the CoDi5.3 element into the two halves found on chromosome 11 and 31.

Furthermore, these two genomic regions contain a group of 5 orthologs of an unknown gene family (see Figure 8 and Additional file 4). The segment containing the two copies located on chromosome 31 and their intergenic region is located less than 1 kb downstream of the CoDi5.3-like element and is highly similar (>97% identity) to the segment containing two of the copies located on chromosome 11 and their intergenic region. The Pt2_50888 gene in fact appears to be the product of recombination between two distinct orthologs as its beginning and downstream region is similar to the Pt2_46949 locus and its end and upstream region appears most similar to the Pt2_46950 and Pt2_50889 loci (Additional file 4). A >7 kb region between the Pt2_46950 locus and the CoDi5.3 segment is also duplicated elsewhere in the genome. These loci therefore provide compelling evidence for TE-mediated recombination events in the *P. tricornutum *genome.

### A high diversity of RT domains from micro-planktonic organisms

**Figure 9:**
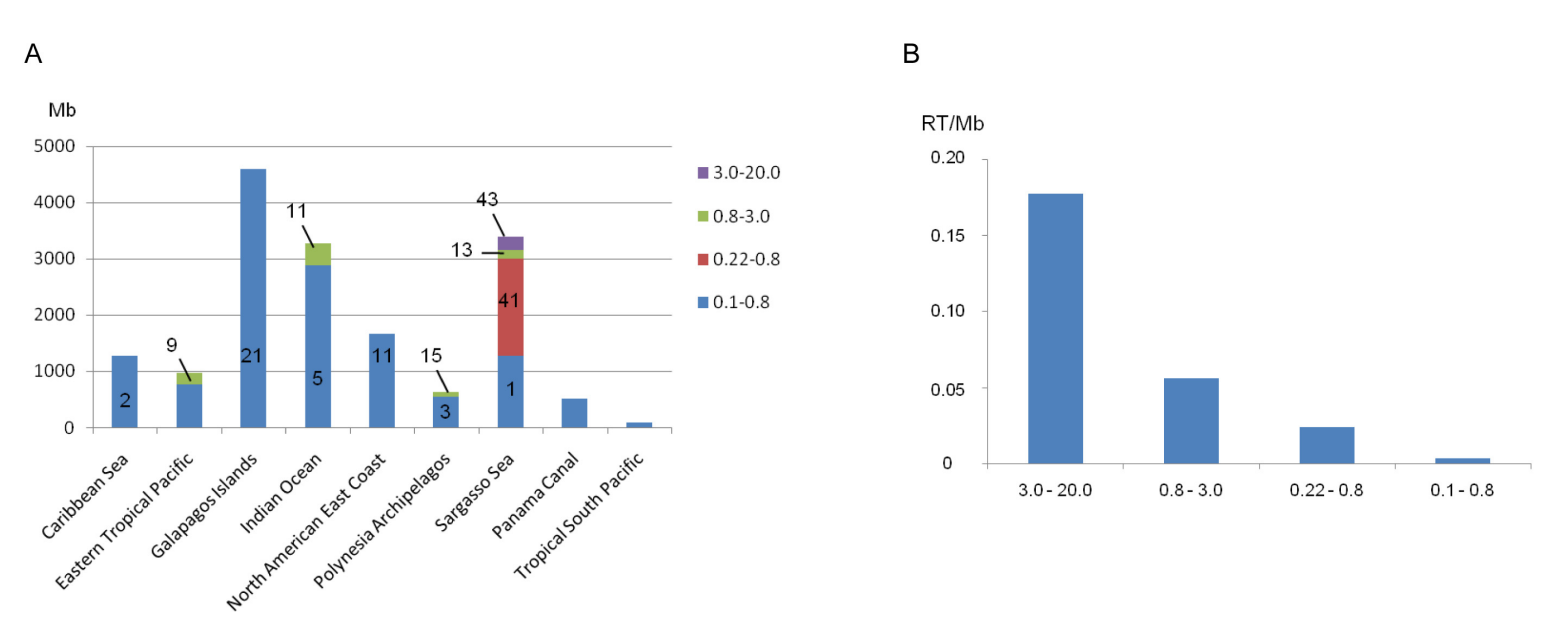
**Distribution of the GOS RT sequences**. (A) Size of dataset in megabases (Mb) for each filter across the different geographic positions examined. Numbers indicate the number of RT hits for each filter. (B) Frequency of RT hits across the different filters.

Very little or no data about RT sequences are available from other eukaryotic clades that include planktonic organisms of ecological importance such as dinoflagellates and coccolithophores. In order to investigate deeper the diversity of LTR-RTs that can be found in planktonic organisms, we used our diatom TE dataset to screen the CAMERA metagenomic database http://camera.calit2.net/, which contains sequences from environmental samples collected during the Global Ocean Sampling (GOS) and Sargasso Sea surveys [29,30]. These sequences are derived from micro-organisms that were trapped on filters of different sizes (0.1-0.8 μm, 0.22-0.8 μm, 0.8-3.0 μm, 3.0-20.0 μm) from the surface water of various parts of the globe including Caribbean Sea, Eastern tropical Pacific, Galapagos Islands, North American East coast, Polynesia Archipelagos, and Sargasso Sea. The size of the database for each filter and at each geographical position is indicated in Figure 9A.

**Figure 10:**
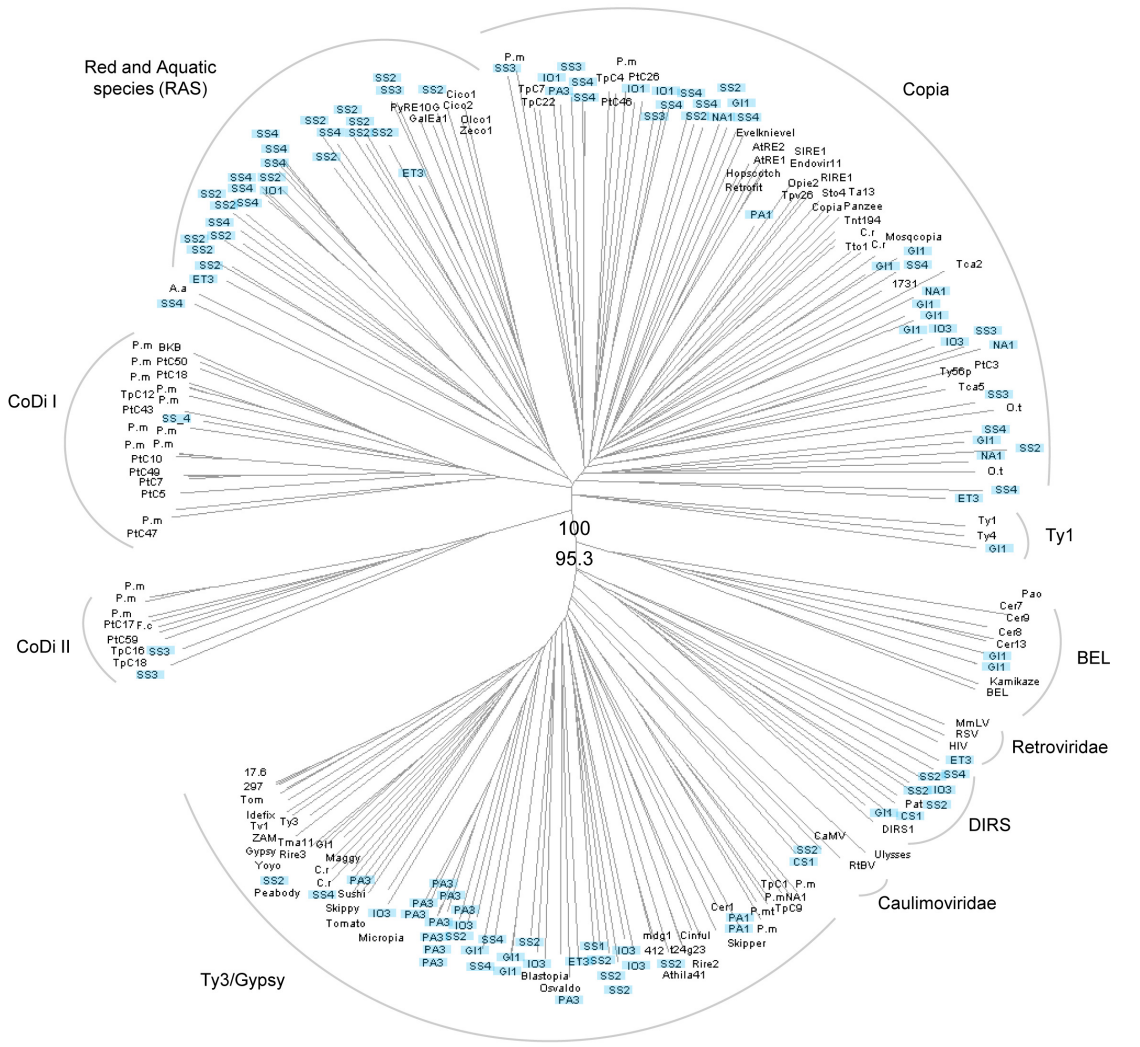
**Phylogenetic tree showing the relationships between the reverse transcriptase domains from the CAMERA database, retroviruses, and LTR retrotransposons**. The tree was constructed with the NJ method using the SplitsTree4 software [59]. The bootstrap values were calculated over 1,000 iterations and are indicated for two basal nodes. GOS sequences are labeled by a two- letter code indicating their geographic provenance: Caribbean Sea (CA), Eastern Tropical Pacific (ET), Galapagos Islands (GI), Indian Ocean (IO), North American East Coast (NA), Polynesia Archipelagos (PA), Sargasso Sea (SS); followed by a number indicating filter size: 0.1-0.8 (1), 0.22-0.8 (2), 0.8-3.0 (3), 3.0-20.0 (4). These labels appear with blue background. Species abbreviations: P. mt (*Pseudonitzschia multistriata*); P. m (*Pseudonitzschia multiseries); *F. c (*Fragilariopsis cylindrus); *C. r (*Chlamydomonas Reinhardtii); *O. t (*Ostreococcus tauri); *A.a (*Aureococcus anophagefferens*).

We queried by BLAST our entire set of RT domains against the CAMERA protein dataset and retrieved a total of 175 subject sequences (Figure 9A), all of which have an LTR-RT for best hit by BLAST comparison with Genbank (data not shown). After normalizing the number of hits from each filter size by its cognate sample size, we observed that the larger the pore size of the filter, the more abundant is the RT domain, with about 0.18 RT domains per Mb of sequence from the 3.0-20.0 μm filters (Figure 9B). A total of 115 of these sequences could be included unambiguously in our RT domain alignment and were used to build a phylogenetic tree in which we also incorporated RT sequences from the green algae *Chlamydomonas reinhardtii *and *Ostreococcus tauri*, the brown alga *Aureococcus anophagefferens*, and the RT domain from the *PyRE10G *element found in the red alga *Porphyra yezoensis *[31] (Figure 10). As expected, we observed an enormous diversity within GOS sequences. It was found that GOS RT domains clustered with all the LTR-RT lineages described here, including the CoDiI and CoDiII lineages. However, RT domains belonging to the Ty3/gypsy, Copia, and the recently identified red/aquatic species (RAS) lineage [32] are the most abundant in the dataset analyzed. We also noticed that the RAS-like lineage appears to be quite a diverse assemblage (Figure 10). These RAS-like elements appear to be the most abundant in the Sargasso Sea samples, especially from the 0.22-0.8 μm filters (data not shown).

## Discussion

In this work, we have identified seven groups of *Ty1/copia*-like LTR-retrotransposons in diatom genomes. Four groups (CoDi1-2-3 and CoDi7) were found only in the *P. tricornutum *genome whereas elements belonging to the CoDi4-5-6 groups were detected in both diatom genomes. The presence of both classes suggests either that they were present in the diatom common ancestor and that the CoDi1-3 groups became extinct in the lineage leading to the centric species *T. pseudonana*, or that representatives of each group have been separately introduced horizontally in pennate and centric diatoms. The topology of the tree presented in Figure 2 shows that CoDi3 and CoDi4 are bootstrap-supported sister groups that share a common ancestor after the separation from CoDi1 and CoDi2. This, together with the fact that we could not detect traces of diverged remnant copies from the CoDi1-3 groups in the *T. pseudonana *genome by BLAST searches (data not shown) favors the horizontal transfer hypothesis to explain the presence of CoDi4 elements in the *T. pseudonana *genome.

*Ty3/gypsy*-like elements were found in the *T. pseudonana *genome but not in the *P. tricornutum *genome. We also identified RT sequences corresponding to *Ty3/gypsy*-like elements from the pennate diatoms *P. multiseries *and *P. multistriata *which clearly cluster with the GyDi elements (Figure 3). Although the number of diatom species for which data is available is low, this suggests that *Ty3/gypsy*-like elements were likely present in the diatom common ancestor, and that these elements have been lost in *P. tricornutum*.

Figure 10 shows the retrotransposon sequences found in the CAMERA dataset. Although the vast majority of the sequences derived from these environmental genomic surveys are of bacterial and archaeal origin [30], the authors counted 69 18S rRNA sequences in the analysis of the Sargasso Sea data [29] and 98 in the GOS sequence collection (Doug Rusch, personal communication). Thus, some small eukaryotes were also present in these datasets. The observed higher abundance of RT domains in the fractions containing the larger cells is consistent with higher relative eukaryote/prokaryote abundance in these samples. The RT sequences studied display a huge diversity including some clustering in the CoDiI and CoDiII lineages, which likely testifies for the presence of diatoms in the samples. The other RT sequences may reflect the diversity of LTR retrotransposons populating the genomes of diverse tiny marine eukaryotes such as green, red, or brown algae, dinoflagellates, haptophytes, or euglenoids. For example, the abundance of RAS-clustering sequences in the Sargasso Sea fractions may be indicative of the presence of red algae, although analysis of these eukaryotic fractions did not reveal a particular abundance of red algae [33]. It will therefore be important to determine which eukaryotic branch or branches the RAS-like sequences collected come from. In addition to the CoDiI, CodiII, and RAS sequences, other discrete clusters shown in Figure 10 are exclusively composed of RT sequences from the CAMERA database and are likely to represent RT domains from organisms for which we have little or no genomic knowledge.

The mutagenic potential of LTR retrotransposons [34] and the effects of their accumulation [35] and recombination [36] together suggest that active retrotransposons may be major contributors to genome diversification. Accumulated data indicates that retrotransposons in plants [37], animals and fungi respond to various forms of stress. It has also been shown in natural wild barley populations living on each side of a canyon that LTR retrotransposon dynamics contribute to genome diversity in response to sharp microclimatic divergence [38]. LTR-RTs are hence thought to play a key role in long term adaptation of natural populations exposed to stress by generating genetic diversity within populations. Evidence presented here suggests that this may also be the case in diatoms. For example, *Blackbeard *is one of the most highly expressed genes in the EST library derived from *P. tricornutum *cells grown under nitrate starvation and *Surcouf *is highly expressed in response to DD treatment (Table 1, Figure 4). If these expression levels correlate with completion of the retrotransposition cycle, which ends with *de novo *insertions, then nitrate starvation, DD exposure, and perhaps other environmental stressors could lead to an increase in genetic diversity in *P. tricornutum*. LTR-RTs may therefore be major drivers of genetic diversity in *P. tricornutum *populations. Although we have not been able to observe *de novo *insertion of *Bkb *or *Scf *elements following stress, this claim is supported by the different insertions that have been observed in *P. tricornutum *accessions isolated from different locations around the world (Figure 6).

The significance of these findings is strengthened by the ecological relevance and common occurrence of stress in marine environments. Nitrogen is the most widespread limiting nutrient for marine phytoplankton [39], and transitions between nitrate starved stratified waters and nitrate replete upwelling conditions are a major influence governing marine diatom population oscillations [40]. Conversely, diatom-derived unsaturated aldehydes can regulate intercellular signalling, stress surveillance, and defence against grazers [41-43]. Diatoms can sense these aldehydes accurately, whereby subthreshold levels serve as an early-warning protective mechanism, and lethal doses initiate a cascade leading to autocatalytic cell death. Activation of *Surcouf *only after exposure to high levels of aldehydes supports a threshold-dependent response in which activation only occurs under acute stress conditions. Furthermore, the fact that significant aldehyde concentrations are only produced by nutrient-stressed and wounded diatoms suggests a possible role in long term adaptation to abiotic and biotic stress [44-47].

## Conclusions

Sexual reproduction in *P. tricornutum *has never been documented. Here, we have seen that *Blackbeard *and *Surcouf *insertions occurred at least a century ago and that both (as well as all other insertions tested; data not shown) have remained in a heterozygous state until now, in accordance with rare or absent meiotic cycles and only limited crossing overs between chromosome pairs in *P. tricornutum*. The maintenance of LTR-RT insertions in a heterozygous state in the *P. tricornutum *genome could increase the genetic variability between haplotypes and hence enhance adaptation capacity to changing environments. Furthermore, the observation that the *Blackbeard *element is hypomethylated in response to nitrate starvation provides a direct link between environmental stress and chromatin remodeling in diatoms. Such phenomena can confer phenotypic plasticity to an individual species, especially if they are heritable, and may be more useful for environmental adaptation than DNA-based modifications, which are irreversible and more likely to lead to speciation and therefore reproductive isolation. It is therefore possible that epigenetic modifications, combined with TE-mediated genomic rearrangements, maintain population diversity in *P. tricornutum*, as opposed to sex-driven chromosomal recombination. The potential capacity of such processes to monitor and to respond rapidly to changing environmental conditions may have contributed to the evolutionary and ecological success of diatoms in contemporary oceans.

## Methods

### Identification of transposable elements

TE complements from the *P. tricornutum *http://genome.jgi-psf.org/Phatr2/Phatr2.home.html and *T. pseudonana *http://genome.jgi-psf.org/Thaps3/Thaps3.home.html nuclear genomes were established by BLAST search [48] using the Repbase library [49] or single TE sequences, redundancy search and search for structural features such as ORFs larger than 1000 amino acids (which are characteristic of LTR-RT) and subsequent BLAST comparison with GenBank. When necessary, full length sequences were determined by examining multi-copy alignment. We then searched for the presence of LTRs upstream and downstream of the DNA sequence corresponding to the ORFs containing a polyprotein. LTR size sometimes varied by a few nucleotides between pairs and the length of the longest LTR is reported in Additional file 1. The target site duplication was examined in the genomic sequence directly flanking the LTRs. The DNA sequences between LTR pairs were translated in order to eventually identify another ORF (denoted ORF1 in Additional file 1) upstream of the ORF containing the polyprotein (ORF2). ORF2 and ORF1 were then submitted to InterProScan http://www.ebi.ac.uk/InterProScan/. The domain composition and order found in ORF2 was established by performing multiple alignments of the putatively active *Ty1/copia*-like elements from *P. tricornutum *and *T. pseudonana *with *Ty1 *from yeast and *Copia *from fruit fly and of the putatively active *Ty3/gypsy*-like elements from *T. pseudonana *with *Gypsy *from fruit fly, and *Ty3 *from yeast.

RT domains from *P. multiseries, P. multistriata, F. cylindrus *[50], *O. tauri, C. reinhardtii, A. anophagefferens *as well as from the GOS and Sargasso Sea metagenomic surveys were found using the RT amino acid sequences from the diatom LTR-RTs identified in this work and a set of RT domains assembled by Gao and collaborators (including elements from the *Ty3/gypsy, Ty1/copia*, DIRS, and BEL groups) as digital probes in BLAST searches [48] directly on the respective cDNA, genomic, and metagenomic databases.

### Classification based on sequence similarity and structural features

We included the *Blackbeard *element in our analysis although it appears to be haplotype-specific and is absent from the final assembly of the *P. tricornutum *genome (see Results). The seven CoDi groups were divided into 26 distinct families on the basis of nucleotide pairwise distances. Further analysis of these elements revealed common structural features that were highly similar within multi-copy families (Additional file 1). Overall, the full length diatom retroelement sequences measure between 5182 bp (TpC22) and 8062 bp (PtC26). LTR length varies from 153 bp to 844 bp in the CoDi4.4 and CoDi3.2 families, respectively, and percent identity between LTR pairs varies from 94% to 100%, meaning that all the elements examined are likely to have inserted relatively recently in their respective genomes. The LTR TG/CA terminal inverted repeat is found in 23 out of 26 families and is missing only in CoDi3.2, CoDi4.2 and CoDi4.3. In some cases, such as the CoDi2.2 family, the terminal repeat is longer and contains up to 8 conserved nucleotides. The duplicated target site or direct repeat (DR) is quite heterogeneous within the groups although the *P. tricornutum *elements from the CoDi5 group consistently differ in a few A/T insertions between duplicates (for which the target site was found). Within the GAG-encoding region of these elements, InterProScan detected tandem CCHC zinc fingers in the elements belonging to the CoDi6.2-6.3-6.4-6.5 families (this domain is commonly found within this region of *Ty1/copia*-like elements).

The selected *Ty3/gypsy*-like elements from *T. pseudonana *represent two rather closely related groups called GyDi1 and GyDi2 (*Ty3/Gypsy *from Diatoms). Structural features of these elements are also shown in Additional file 1. We submitted one element from each family to GenBank (accession numbers are shown in Additional file 1).

### Phylogenetic analysis

Multiple alignments were performed with the CLUSTALW program [51]. Genetic distances were calculated with the Poisson correction method [52] for amino acid sequences and phylogenetic trees were constructed with the Neighbor-Joining method [53]. These evolutionary analyses were performed with the MEGA4 and SplitsTree4 platforms [54,55].

In addition to the RT sequences identified in this work, phylogenetic trees presented in Figure 3 includes RT domains from Ty1/Copia, Ty3/Gypsy, DIRS and BEL LTR-RT lineages [56], as well as RT sequences from the Retroviridae human immunodeficiency virus type 1 (HIV), Rous sarcoma virus (RSV), and moloney murine leukaemia virus (MmLV) and from the Caulimoviridae Cauliflower mosaic virus (CaMV) and Rice tungro bacilliform virus (RtBV).

In addition to the sequences used in Figure 3 and 115 RT sequences from the CAMERA metagenomic database http://camera.calit2.net/, Figure 5 is built from a CLUSTALW alignment including also four RT sequences from *C. reinhardtii*, two from the *O. tauri*, one RT sequence from *A. anophagefferens *http://www.jgi.doe.gov/, and the RT sequence of the *PyRE10G *element from *P. yezoensis *(AB286055). For all phylogenetic analysis, the residues used were a modification of those originally identified by Toh et al. [57,58] in retroviral, human hepatitis B virus (HBV), cauliflower mosaic virus (CaMV), and several retrotransposon sequences from Drosophila [10].

### Cell culture and accessions

Axenic cultures of *P. tricornutum *Bohlin clone Pt1 8.6 (CCMP2561) were obtained from the culture collection of the Provasoli-Guillard National Center for Culture of Marine Phytoplankton, Bigelow Laboratory for Ocean Sciences, USA. Cultures were grown in f/2 medium [59] made with 0.2-μm-filtered and autoclaved local seawater supplemented with f/2 vitamins and inorganic nutrients (filter sterilized and added after autoclaving). Cultures were incubated at 18°C under cool white fluorescent lights at approximately 75 μmol.m^-2^.s^-1 ^constant light and maintained in exponential phase in semi-continuous batch cultures. Sterility was monitored by occasional inoculation into peptone-enriched media to check for bacterial growth [60].

In order to evaluate the effect of nitrate stress on *Blackbeard *mRNA levels, cells were transferred to media modified with 50 μM NO_3_^- ^and maintained in exponential phase in semi-continuous batch cultures. Samples were collected after 24 hrs and after 2 weeks exposure to nitrogen limitation. In order to evaluate the effect of diatom-derived reactive aldehydes on *Surcouf *transcript abundance, 2 liters of exponential *P. tricornutum *culture was treated with 2 μg/mL (2E,4EZ )-decadienal (DD) and control culture was treated with equivalent volume of methanol (DD solvent). Samples of 250 mL were collected in the indicated time points (0, 2, 6, 30, 96 hr) after exposure to DD treatment. (2E,4*E/Z *)-decadienal (DD) was obtained from Acros Organics USA. DD was dissolved in methanol, and concentrations were determined by measuring absorption at the lambda max for DD of 274 nm, using a Hewlett-Packard 8453 spectrophotometer. Diatom cells were harvested by centrifugation for 15 min at 3,000 *g*, washed with 12 mL of PBS, aliquoted into 2 mL Eppendorf tubes, and pelleted for 3 min at 10,000 *g*. Cell pellets were frozen instantly in liquid nitrogen and stored at -80°C before proceeding with RNA extraction.

The original sampling location of the *P. tricornutum *accessions Pt1-10 have been recently described in [27]. We recently obtained three additional *P. tricornutum *accessions that we included for our SSAP analysis. Pt11 and Pt13 were sampled in 2008, respectively, in the Gulf of Naples and the Gulf of Salerno, Italy. Pt12 was obtained from the Roscoff culture collection.

### SSAP

SSAP experiments were conducted as previously described [61]. Genomic DNA (500 ng) was digested with MspI and ligated to an MspI adaptor obtained by the annealing of two primers (Adap-MspI-C: 5'-CGT TCT AGA CTC ATC-3' and Adap-MspI-L: 5'-GAC GAT GAG TCT AGA A-3'). SSAP amplification was done by using a non labeled adaptor primer Msp1 (5'-GAT GAG TCT AGA ACG GC-3') and one of the following ^33^P-labelled LTR primers (*Bkb, Scf *and PtC34). Amplified products were separated on 6% denaturing polyacrylamide gels and exposed after drying to Kodak BioMax XAR films (Carestream Health Inc, Rochester). List of LTR primers: *Bkb*-Rev: 5'-ACG ATA ACC GAC CAG AAT CG-3' *Scf*-Rev: 5'-CCC GAA AAA CAT TGC CTC TA-3' PtC34-Rev: 5'-ATC GGA TCC AGG ACT TTG TG-3'

### RNA purification and reverse transcription

mRNA levels of *Blackbeard *and *Surcouf *were analyzed using q-RT-PCR from triplicate samples collected from biological replicates of nitrate starved or DD-treated exponential grown cultures. Total RNA was extracted from approximately 10^8 ^cells using TRIzol Reagent (Invitrogen) and contaminating DNA was removed with TURBO DNase via treatment (Ambion), both according to manufacturer's protocols. RNA was then reverse transcribed into first strand cDNA with the SuperScript™ III First-Strand Synthesis System for RT-PCR (Invitrogen) using oligo-dT primers. Gene transcription was measured using the Brilliant^® ^SYBR^® ^Green QPCR Core Reagent Kit and the Stratagene MX3000P QPCR machine (Stratagene). Primers used for real-time PCR were *Surcouf *Fwd, 5'-CGA CCA CCG GCA TAC TTA TT-3', *Surcouf *Rev 5'-GGT TGT ACC GCA AGG CTA TG-3', *Blackbeard *Fwd 5'-GTG TTC TTG CTG CAA ATG GA-3', *Blackbeard *Rev 5'-ATT CAT CGG GGT CAC CAA TA-3', 18S rDNA Fwd 5'-CAT CCT TGG GTG GAA TCA GT-3' and 18S rDNA 5'-TGC GCA AAC CAA CAA AAT AG-3'. Additional primer sets were designed for Histone H4 and for TBP (TATA box binding protein) which served as a housekeeping gene for normalizing expression of the target gene [62]. For each treatment, we evaluated each of the housekeeping genes and selected the one that showed the least amount of variation across conditions.

### GUS assay

The pLTRbkb-GUS-FcpA plasmid was constructed from the FcpBp-GUS-FcpA vector [63] in which the FcpB promoter has been removed by KpnI/SalI digestion and replaced by ligation with a PCR fragment corresponding to *Blackbeard *LTR amplified using the Fwd 5'-CTT AGT GGT ACC TAG AAA AAC CCC ACG TCA AGC-3' and Rev 5'-CTT AGT GTC GACGAT AAA CTA GAA AAC TGC AAC GAT AAC-3' and digested with KpnI/SalI. The pLTRbkb-GUS-FcpA vector was introduced into *P. tricornutum *by microparticle bombardment using a Biolistic PDS-1000/He Particle Delivery System (Bio-Rad, Hercules, CA, USA) as described by Falciatore et al. [63].

For β-glucuronidase (GUS) assays, 7 colonies carrying the pLTRbkb-GUS-FcpA construct were grown to mid-log phase in media containing 50 or 882 μM NO_3_^-^. Two weeks after cells were transferred to 50 μM NO3-, 20 ml cultures were collected by centrifugation at 3,800 rpm for 15 min at 4°C and resuspended in 120 μl freshly prepared GUS extraction buffer (50 mM NaPO_4 _pH 7.0, 10 mM β-mercaptoethanol, 0.1% Triton X-100), twice frozen in liquid nitrogen and thawed at 37°C, and finally centrifuged at 12,000 rpm for 5 min at 4°C. Soluble proteins were quantified with the Bio-Rad Protein Assay. The fresh extracts were used for spectrophotometric GUS assays performed by incubating at least 10 μg of total protein extract with the GUS enzyme substrate p-nitrophenyl glucuronide (PNPG) at a final concentration of 1 mM, in a total reaction volume of 1 ml. After a one hour incubation at 37°C, the colorimetric reaction was stopped by adding 0.4 ml 2.5 M 2-amino-2-methyl-1,3-propandiol and the absorbance measured at 415 nm. The enzymatic GUS activity was calculated on the base of the O.D. recorded and the molar extinction coefficient of the GUS substrate p-nitrophenol. One unit is defined as the quantity of enzyme that produces one nanomole of product in one minute at 37°C [64].

### McrPCR

*P. tricornutum *cells grown were grown as described above under normal and nitrate-limited conditions for two weeks. DNA and RNA were extracted from 20 mL of culture for each condition. After cDNA synthesis from RNA samples (as described above) *Blackbeard *expression was verified by semi-quantitative RT PCR using the primers used for Q-PCR (see above) and primers amplifying the H4 and RPS housekeeping genes as controls [62]. For McrPCR, 1 μg of DNA from each sample was incubated for 1 hour at 37°C with 20 units McrBC endonuclease supplemented with 100 μg/ml bovine serum albumin and 1 mM guanosine triphosphate. Negative controls were obtained with the same experimental procedure but replacing guanosine triphosphate with water. The enzyme was subsequently inactivated by incubation at 65°C for 10 minutes. Digestion efficiency of the *Blackbeard *locus was measured by semi-quantitative PCR using forward genomic primer -AAT ATT GGT CTT CGG CAA CG-3' and the *Blackbeard*-specific reverse primer 5'-GCT TCC GTC AAA CAC TCA CA-3' and we used the primers amplifying the H4 and RPS genes as controls (see above).

### PCR haplotype/accession analysis

Polymerase chain reactions were performed using template DNA extracted from cultures of the ten different *P. tricornutum *accessions (see previous). The primers used to assess the presence of the two different haplotypes at the *Blackbeard *locus in DNA extracts from the ten accessions were the genomic Fwd 5'-AAT ATT GGT CTT CGG CAA CG-3' paired with the genomic Rev 5'-TTT GAC CCT ATT GGC TAC CG-3' or paired with the *Blackbeard*-specific Rev 5'-GCT TCC GTC AAA CAC TCA CA-3'. The primers used to assess the presence of the two different haplotypes at the *Surcouf *locus were the genomic Fwd 5'-TGT CTA TTG ACA TTT TGG AAG GTG-3'paired with the genomic Rev 5'-AGA TTC ATC AAT GGA TCA TCT CTC-3' or paired with the *Surcouf*-specific Rev 5'-GGG TAC CTG CTC CAT ATG TAG GTT-3'. Additional primer sets were designed for the other insertions analyzed.

## Authors' contributions

FM, AEA and CB planed the experiments and wrote the manuscript. AEA carried out the QPCR experiments in response to nitrate starvation. CM and MAG carried out the SSAP experiments and contributed to the final version of the manuscript. AV carried out the QPCR experiments in response to (2E, 4EZ )-decadienal and contributed to the final version of the manuscript. HH carried out the GUS assays and participated in the analysis of polymorphic bands obtained by SSAP. KJ carried out the RepeatMasker analysis and contributed to the final version of the manuscript. FM carried out all other experiments and analyses. All authors read and approved the final manuscript.

## Supplementary Material

Additional file 1 **List of putatively active LTR-RTs found in diatom genomes**. Classification, structural features, and accession numbers of the putatively active LTR-RTs identified in the *P. tricornutum *and *T. pseudonana *genomes. Click here for file

Additional file 2 **Polymorphism generated by TE insertions across *P. tricornutum *accessions**. Distribution of polymorphic bands obtained by SSAP experiments (with BKB, SCF, and PtC34) across 13 *P. tricornutum *accessions and positions of the corresponding sequences in the Pt1 genome when occurring only once (otherwise, we indicated the nature of the repeat sequenced). Click here for file

Additional file 3 **Haplotype specificity of *Blackbeard *insertion**. (A) Close up on the dot-plot comparison (window size: 11) of two consensus sequences of the *Blackbeard *insertion locus retrieved with the help of the Stanford Human Genome Center. (B) Schematic view of the two haplotypes observed at the *Blackbeard *insertion locus in the *P. tricornutum *genome. Haplotype "a" corresponds to the one found in the final version of the *P. tricornutum *genome assembly http://genome.jgi-psf.org/Phatr2/Phatr2.home.html and haplotype "b" corresponds to the empty allele. Sequence of the target site duplication upon *Blackbeard *insertion (TTCC) is shown in red. Green arrows represent gene models neighbouring this locus. Click here for file

Additional file 4 **Pt2_50588 consists in a recombination product**. Close up on the sequence alignment of the Pt2_50588 orthologs at the level of the transition between higher similarities of Pt2_50588 with Pt2_46949/Pt2_46953 (highlighted in blue) and with Pt2_46950/Pt2_50589 (highlighted in red). Click here for file
