# Supplementary figures and images for: Potential impact of stress activated retrotransposons on genome evolution in a marine diatom
Source: BMC Genomics. 2009 Dec 22;10:624. doi: 10.1186/1471-2164-10-624 (PMC2806351; doi:10.1186/1471-2164-10-624)

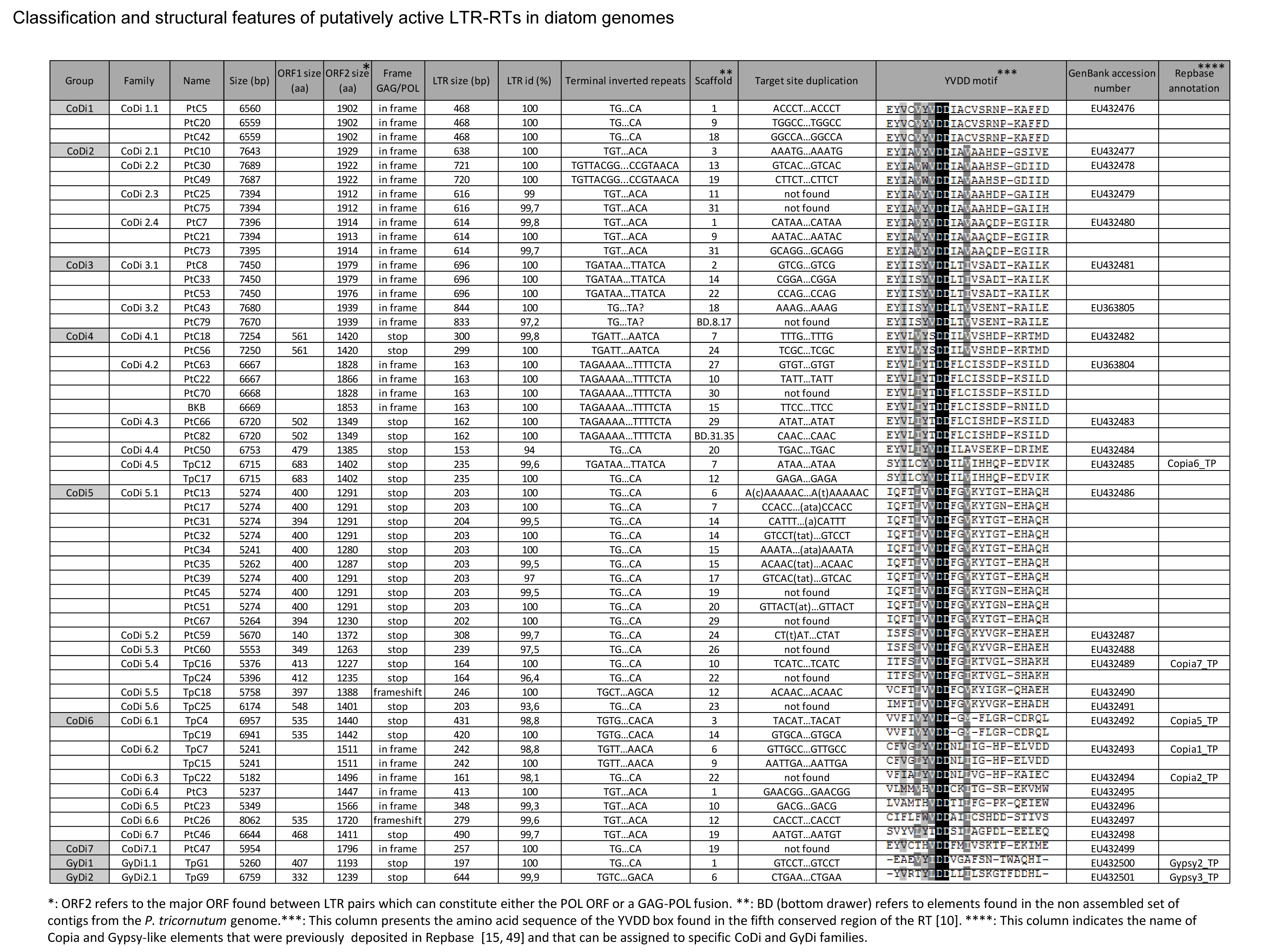

Supplement: Additional file 1 — List of putatively active LTR-RTs found in diatom genomes. Classification, structural features, and accession numbers of the putatively active LTR-RTs identified in the P. tricornutum and T. pseudonana genomes. [file 1471-2164-10-624-S1.TIFF]

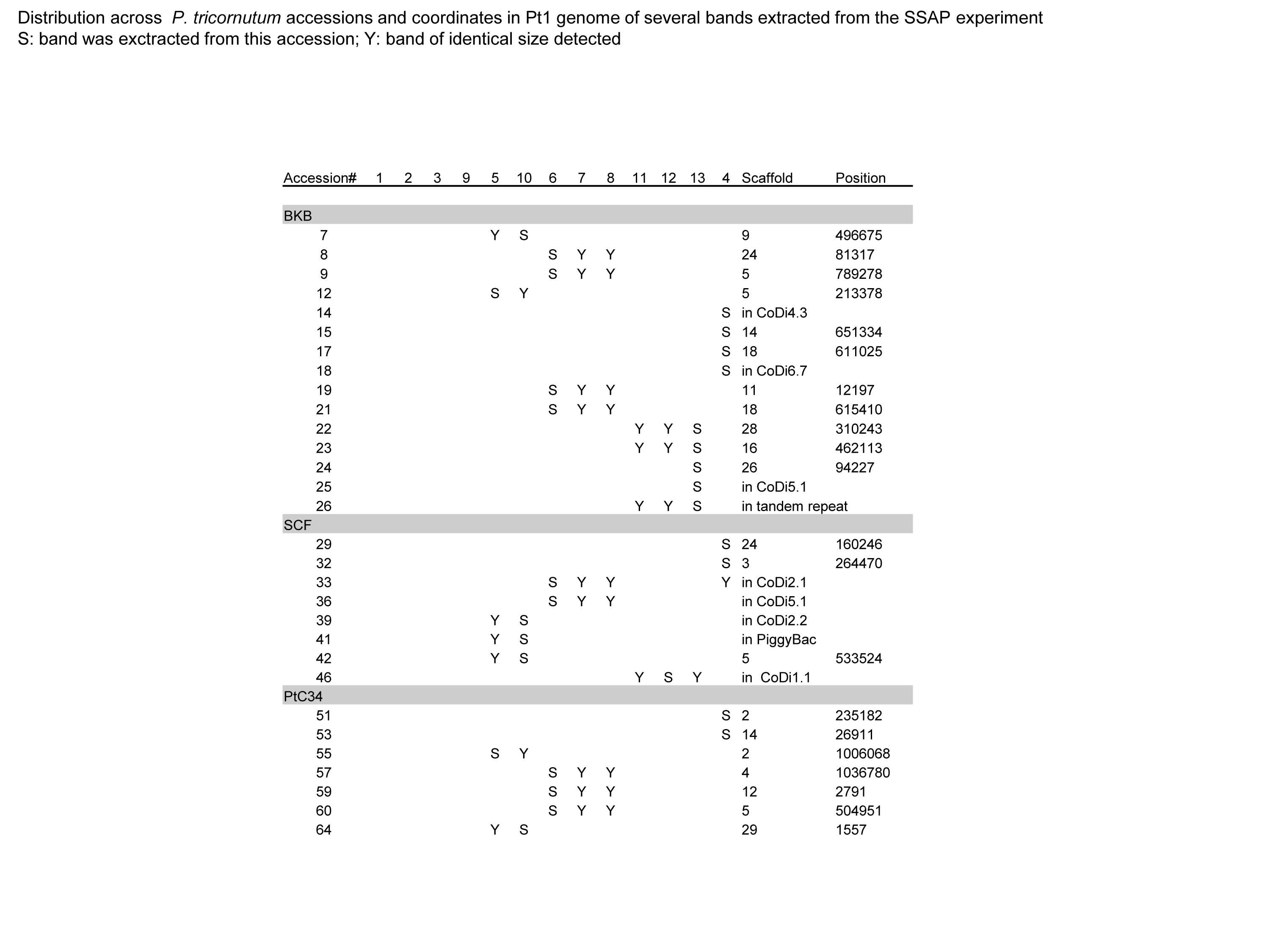

Supplement: Additional file 2 — Polymorphism generated by TE insertions across P. tricornutum accessions. Distribution of polymorphic bands obtained by SSAP experiments (with BKB, SCF, and PtC34) across 13 P. tricornutum accessions and positions of the corresponding sequences in the Pt1 genome when occurring only once (otherwise, we indicated the nature of the repeat sequenced). [file 1471-2164-10-624-S2.TIFF]

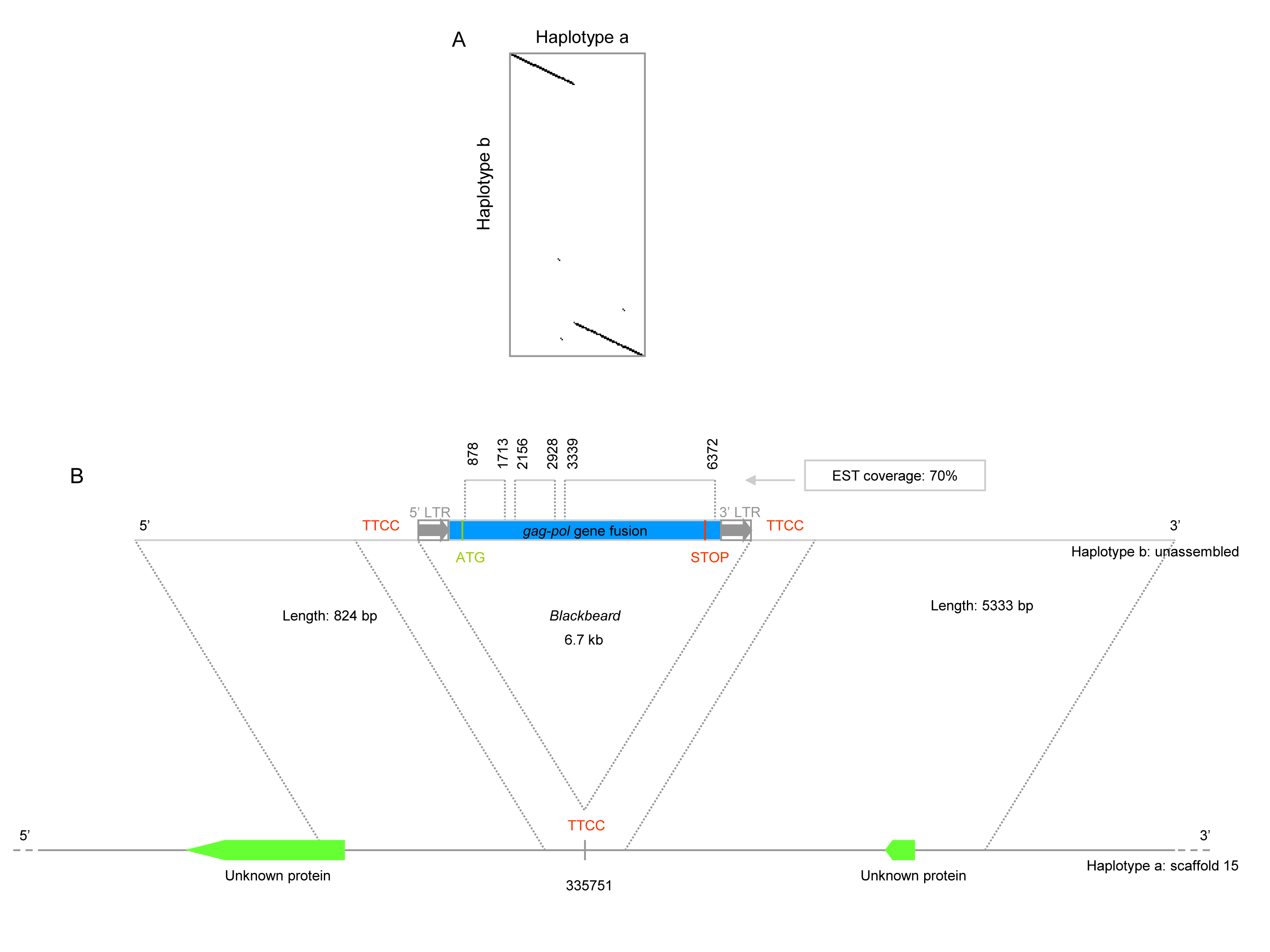

Supplement: Additional file 3 — Haplotype specificity of Blackbeard insertion. (A) Close up on the dot-plot comparison (window size: 11) of two consensus sequences of the Blackbeard insertion locus retrieved with the help of the Stanford Human Genome Center. (B) Schematic view of the two haplotypes observed at the Blackbeard insertion locus in the P. tricornutum genome. Haplotype "a" corresponds to the one found in the final version of the P. tricornutum genome assembly http://genome.jgi-psf.org/Phatr2/Phatr2.home.html and haplotype "b" corresponds to the empty allele. Sequence of the target site duplication upon Blackbeard insertion (TTCC) is shown in red. Green arrows represent gene models neighbouring this locus. [file 1471-2164-10-624-S3.TIFF]

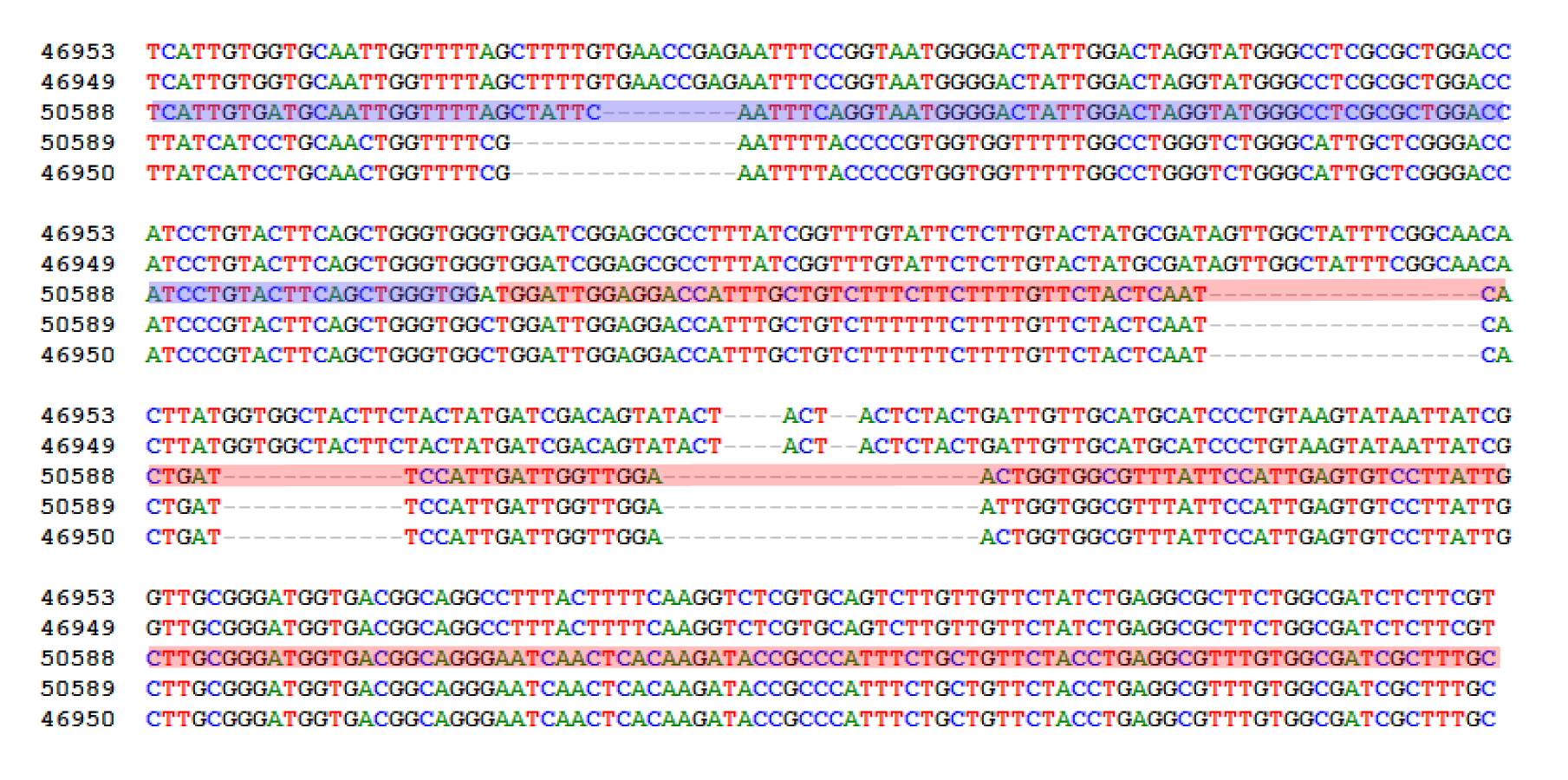

Supplement: Additional file 4 — Pt2_50588 consists in a recombination product. Close up on the sequence alignment of the Pt2_50588 orthologs at the level of the transition between higher similarities of Pt2_50588 with Pt2_46949/Pt2_46953 (highlighted in blue) and with Pt2_46950/Pt2_50589 (highlighted in red). [file 1471-2164-10-624-S4.TIFF]
